# Emergency Appliances on Railways

**Published:** 1889-01

**Authors:** 


### *EMERGENCY APPLIANCES ON RAILWAYS.*

Anything that will contribute to the welfare and safety of the traveling public, and of the employés of the great transportation companies of our country, is usually readily adopted by the companies, when properly presented to them. The number of accidents is comparatively small, but the aggregate of damages paid by railway companies to injured employés and passengers is enormous. The loss of life and limb is much reduced by prompt arrest of hæmorrhage and thorough antiseptic occlusion of the contused wounds that usually result from railway accidents. These ends might be promoted by having in every railway coach and station sealed packages of hæmostatic appliances and antiseptic dressings, wrapped in covers on which are printed pictured and verbal directions for their use. At the termini of each road, instruction in the use of these dressings and appliances could be easily given the train men by skilled persons. The unions and benevolent societies are directly interested in this matter. The train men are of unusual intelligence, and the interest they would take in such instruction would be increased by the knowledge that sooner or later they might very likely need the assistance they are taught to give. Many more employés than passengers are injured. With the former, injuries to the hand are the most frequent. It is well-known that the most terrible contusions of this extremity are rapidly repaired under antiseptic dressings, and the organ restored to a very fair degree of usefulness. Thus the State may be saved the care and expense of a useless, and perhaps a vicious citizen. It would be well to prepare such packages and place them in those stations and switch-houses about which statistics show that an unusual number of accidents are accustomed to happen. In this way their usefulness could be demonstrated, and the subject brought to the notice of those most concerned.

